# Ferroptosis in compromised bone healing after trauma: from the iron/ROS microenvironment to non-union and bone defect reconstruction

**DOI:** 10.3389/fmed.2026.1803197

**Published:** 2026-04-10

**Authors:** Chaowei Ouyang, Dandan Zhang, Changbin Lei

**Affiliations:** 1 Department of Orthopedic Surgery, West China Hospital, Sichuan University, Chengdu, China; 2 West China School of Nursing, Sichuan University, Chengdu, China; 3 Trauma Center, West China Hospital, Sichuan University, Chengdu, China

**Keywords:** biomaterials, bone healing, bone reconstruction, ferroptosis, GPX4, iron metabolism, lipid peroxidation

## Abstract

Compromised bone healing—encompassing osteoporotic fractures, large segmental defects requiring induced membrane technique, infected bone defects, and non-union—remains a significant clinical challenge. Although mechanical, vascular, and infectious factors are well-recognized contributors, the molecular mechanisms underlying cellular dysfunction in these contexts remain incompletely understood. Ferroptosis, an iron-dependent form of regulated cell death driven by lipid peroxidation, has recently emerged as a potential contributor to impaired bone regeneration. This narrative review synthesizes current evidence on ferroptosis in compromised bone healing. We first outline the core biochemistry of ferroptosis, focusing on iron metabolism, lipid peroxidation, and the GPX4/Nrf2 antioxidant axis. We then characterize the post-traumatic microenvironment as a ferroptosis-permissive niche, where hematoma-derived iron release, inflammatory ROS generation, and ischemia-reperfusion injury converge to create conditions favoring ferroptotic cell death. We subsequently review original studies linking ferroptosis to specific clinical phenotypes, highlighting context-dependent mechanisms: NCOA4-mediated ferritinophagy in osteoporotic and smoking-associated contexts, and macrophage ferroptosis with TNF-α-mediated paracrine suppression of osteogenesis in infected defects. Finally, we discuss therapeutic strategies organized by intervention module—iron homeostasis restoration, lipid peroxidation suppression, GPX4/Nrf2 reinforcement, and multifunctional biomaterial platforms—and articulate design principles for integrating ferroptosis modulation into reconstruction workflows. Ferroptosis represents a mechanistically coherent and therapeutically addressable target in compromised bone healing. Translating these insights into clinical practice will require human tissue validation, optimized intervention timing, and prospective trials with reconstruction-relevant endpoints.

## 1 Introduction

Fracture healing is a remarkably orchestrated biological process that recapitulates aspects of embryonic skeletal development, requiring the precise spatiotemporal coordination of inflammation, angiogenesis, osteogenesis, and remodeling (1–3). Under optimal conditions, the majority of fractures heal uneventfully within weeks to months. However, a substantial subset of fractures—estimated at 5%–10% of all cases—progresses toward compromised healing outcomes, including delayed union, non-union, and critical-sized bone defects that fail to regenerate without surgical intervention (4–7). These conditions impose considerable clinical and socioeconomic burdens, often necessitating multiple revision surgeries, prolonged immobilization, and substantial healthcare expenditures (8, 9). Despite significant advances in fixation technology, biological augmentation, and reconstructive techniques over the past decades, the fundamental biological barriers underlying compromised healing remain incompletely understood and insufficiently targeted therapeutically (10, 11).

The pathophysiology of compromised bone healing is multifactorial, encompassing mechanical instability, inadequate vascularization, impaired osteogenic cell function, persistent inflammation, and infection (12–14). A recurring theme across these diverse etiologies is the presence of a hostile microenvironment characterized by excessive oxidative stress, dysregulated iron metabolism, and sustained inflammatory signaling (15–17). Traumatic injury initiates a cascade of events—hemorrhage and hematoma formation, erythrocyte lysis and hemoglobin release, ischemia–reperfusion injury, and inflammatory cell infiltration—that collectively generate substantial quantities of reactive oxygen species (ROS) and liberate catalytically active iron into the local tissue milieu (18–20). While transient oxidative signaling and controlled iron availability are essential for normal healing responses, their dysregulation creates a biochemically permissive environment for pathological cell death and tissue damage (21, 22).

Ferroptosis, first characterized by Dixon et al. in, is a distinct form of regulated cell death driven by iron-dependent phospholipid peroxidation (23). Unlike apoptosis, necroptosis, or pyroptosis, ferroptosis is defined by the catastrophic accumulation of lipid hydroperoxides in cellular membranes, leading to membrane rupture and cell demise (24–26). The core biochemistry of ferroptosis involves three interconnected modules: labile iron that catalyzes Fenton-type radical generation, polyunsaturated fatty acid (PUFA)-containing phospholipids that serve as peroxidation substrates, and antioxidant defense systems—most notably the System Xc^–^/glutathione (GSH)/glutathione peroxidase 4 (GPX4) axis—that counteract lipid peroxidation (27–29). When the balance tips toward pro-ferroptotic conditions, cells exceeding their lipid peroxidation threshold undergo ferroptotic death. Over the past decade, ferroptosis has been implicated in a broad spectrum of pathologies, including neurodegeneration, ischemia–reperfusion injury, cancer, and, increasingly, musculoskeletal disorders (30–34).

In the context of bone biology, ferroptosis has emerged as a significant contributor to osteoblast and osteocyte dysfunction, with accumulating evidence linking ferroptotic cell death to osteoporosis, glucocorticoid-induced osteonecrosis, and implant-associated osteolysis (35–38). More recently, attention has turned to the potential role of ferroptosis in fracture healing and bone regeneration. The post-traumatic microenvironment—with its characteristic iron overload, ROS excess, and inflammatory perturbation—appears to create conditions that prime osteogenic lineage cells, mesenchymal stromal/stem cells (MSCs), and immune cells toward ferroptotic vulnerability (39). If ferroptosis depletes critical cellular effectors during the early inflammatory and reparative phases, the downstream consequences may include impaired callus formation, deficient vascularization, and ultimately, failure to achieve bony union.

Despite growing mechanistic interest, the ferroptosis field in bone healing research remains fragmented. Trauma surgeons and reconstructive specialists typically frame compromised healing in terms of mechanical stability, infection control, and vascular supply, while ferroptosis researchers often approach bone from a cell biology perspective without explicit consideration of surgical workflows, staged reconstruction, or clinical phenotype heterogeneity (40). This disconnect limits the translational impact of ferroptosis research and obscures opportunities for clinically actionable intervention. Furthermore, much of the existing literature on ferroptosis in bone derives from osteoporosis models or *in vitro* osteoblast studies, with comparatively limited direct investigation of fracture healing, non-union, infected bone defects, or reconstruction-specific contexts such as the induced membrane technique (IMT) (41, 42).

This review synthesizes current knowledge on ferroptosis in compromised bone healing, examining its biochemical basis, the ferroptosis-permissive post-traumatic microenvironment, and evidence across clinical phenotypes including osteoporotic fractures, induced membrane technique, infected bone defects, and non-union. Translational opportunities for ferroptosis-targeted biomaterial strategies compatible with reconstruction workflows are also discussed.

## 2 Ferroptosis: core biochemistry and regulatory networks relevant to bone repair

### 2.1 Defining features and molecular mechanisms of ferroptosis

Ferroptosis is a form of regulated cell death mechanistically distinct from apoptosis, necroptosis, and autophagic cell death (23, 24). Its defining biochemical feature is the iron-catalyzed peroxidation of polyunsaturated fatty acid (PUFA)-containing phospholipids within cellular membranes, leading to catastrophic membrane destabilization and cell demise (43). Morphologically, ferroptotic cells exhibit characteristic mitochondrial shrinkage with increased membrane density and reduced cristae, while the nucleus remains intact without apoptotic chromatin condensation (44).

At its core, the ferroptotic cascade follows a clear causal logic: catalytically active ferrous iron (Fe^2+^) drives Fenton-type reactions that generate hydroxyl radicals (⋅OH) and other reactive oxygen species; these radicals attack polyunsaturated fatty acid (PUFA)-containing phospholipids in cellular membranes, initiating lipid peroxidation chain reactions; and when the resulting lipid hydroperoxides accumulate beyond the detoxification capacity of the cell’s antioxidant defenses—most notably glutathione peroxidase 4 (GPX4)—membrane integrity is catastrophically compromised, leading to cell death (45). This mechanistic sequence defines three interconnected regulatory modules whose balance determines cellular susceptibility to ferroptosis: (i) iron metabolism, which controls the availability of catalytic Fe^2+^; (ii) lipid composition, which determines the abundance of peroxidation-susceptible substrates; and (iii) antioxidant defense capacity, principally the System Xc^–^/GSH/GPX4 axis, which counteracts lipid peroxidation (27–29, 45). Regarding the first module, iron exerts its pro-death function primarily through catalyzing Fenton reactions (Fe^2+^ + H_2_O_2_ → Fe^3+^ + OH + OH^–^), in which ferrous iron reacts with hydrogen peroxide to generate highly reactive hydroxyl radicals that initiate lipid radical chain reactions (46, 47). The intracellular labile iron pool is regulated by transferrin receptor 1 (TfR1) for uptake, ferritin for storage, and ferroportin for export (48, 49). Notably, ferritinophagy—the NCOA4-mediated autophagic degradation of ferritin—can liberate stored iron and amplify ferroptotic vulnerability (50, 51). In traumatic contexts, heme released from damaged erythrocytes represents an additional iron source; heme oxygenase-1 (HO-1) degrades heme to release free iron, a process that may be cytoprotective or pro-ferroptotic depending on downstream iron-buffering capacity (52, 53).

The second determinant is membrane lipid composition. PUFAs are highly vulnerable to peroxidation due to their bis-allylic hydrogen atoms, generating lipid hydroperoxides and reactive aldehydes such as 4-hydroxynonenal (4-HNE) and malondialdehyde (MDA) (54–56). Critically, these lipid hydroperoxides are not inert end-products; they can abstract hydrogen atoms from adjacent PUFAs, propagating a self-amplifying chain reaction that progressively expands membrane damage until antioxidant defenses are overwhelmed (54–56). Acyl-CoA synthetase long-chain family member 4 (ACSL4) sensitizes cells to ferroptosis by incorporating arachidonic acid into membrane phospholipids; accordingly, ACSL4 expression correlates strongly with ferroptotic vulnerability (57, 58).

The third and most extensively studied module is the antioxidant defense system. The canonical pathway centers on glutathione peroxidase 4 (GPX4), a selenoenzyme that reduces phospholipid hydroperoxides to non-toxic alcohols (59, 60). GPX4 activity depends on reduced glutathione (GSH), whose synthesis requires cysteine imported via the System Xc^–^ cystine/glutamate antiporter (61). Pharmacological inhibition of System Xc^–^ (erastin) or GPX4 (RSL3) triggers ferroptosis by depleting this axis (61). Beyond this canonical pathway, GPX4-independent mechanisms including the FSP1/CoQ_10_ axis and the GCH1/tetrahydrobiopterin pathway provide additional protection (62–65).

Nuclear factor erythroid 2-related factor 2 (Nrf2) orchestrates the transcriptional regulation of multiple ferroptosis-related genes, including those involved in GSH synthesis, iron handling, and NADPH regeneration (66). Nrf2 activation generally exerts protective effects, though its target gene HO-1 presents context-dependent duality: while generating antioxidant products, HO-1 (67) also releases free iron from heme, potentially promoting ferroptosis when iron sequestration capacity is exceeded (68, 69). This nuance is particularly relevant to trauma contexts where heme burden may be substantial.

### 2.2 Ferroptosis in bone cells and practical readouts for skeletal research

The principal cell types involved in bone metabolism—bone marrow-derived mesenchymal stem cells (BMSCs), osteoblasts, osteoclasts, and osteocytes—are all susceptible to ferroptotic cell death under appropriate conditions (70, 71). BMSCs, as the common progenitors of osteoblasts and other mesenchymal lineage cells, occupy a critical upstream position in bone regeneration. Iron overload has been shown to induce aberrant differentiation and senescence of BMSCs, shifting their lineage commitment away from osteogenesis toward adipogenesis (72, 73). Furthermore, BMSCs exposed to ferroptosis-inducing conditions exhibit suppressed proliferative and osteogenic capacity, with decreased GSH levels, elevated lipid peroxidation, and reduced GPX4 expression (72, 73). Because BMSCs are the primary cellular reservoir for fracture repair—their recruitment, survival, and osteogenic differentiation are prerequisites for callus formation—ferroptotic attrition of this progenitor population may fundamentally compromise the regenerative response. This vulnerability is particularly relevant in osteoporotic and iron-overload conditions where BMSCs face compounded oxidative stress, a theme explored further in section “4.1 Osteoporotic fractures and bone defects.” Osteoblasts, the differentiated progeny of BMSCs responsible for bone matrix synthesis and mineralization, undergo ferroptosis in response to high glucose, glucocorticoid excess, iron overload, and inflammatory cytokine exposure, with decreased GPX4/SLC7A11 expression, elevated ACSL4, and increased lipid peroxidation products (74–76). Osteoclasts, while primarily studied in the context of ferroptosis-induced modulation of resorptive activity, may also experience altered function under conditions of iron dysregulation and oxidative stress (74–76). Osteocytes, the most abundant bone cells and principal mechanosensors, are similarly vulnerable to ferroptosis; given their role in coordinating bone remodeling through RANKL/OPG signaling, osteocyte ferroptosis may disrupt the balance between bone formation and resorption, affecting both osteoblast and osteoclast function (38).

For orthopedic research, convincing ferroptosis evidence requires multiple orthogonal readouts (77, 78). Functional criteria are paramount: ferroptosis inhibitors (ferrostatin-1, liproxstatin-1, deferoxamine) should rescue the phenotype, while inducers (erastin, RSL3) should reproduce it (79). Molecular signatures include decreased GPX4/SLC7A11 and increased ACSL4 expression (80). Lipid peroxidation can be assessed via C11-BODIPY fluorescent probe, 4-HNE immunostaining, or MDA quantification (81, 82). Iron accumulation is visualized by Prussian blue staining or Fe^2+^-selective probes (83). Transmission electron microscopy revealing characteristic mitochondrial morphology provides ultrastructural confirmation.

A critical consideration is temporal heterogeneity: a marker indicating ferroptotic activity in the early inflammatory phase may have different implications than the same finding during callus maturation. For example, transient GPX4 suppression during acute inflammation may be recoverable, while persistent suppression could signify a pathological trajectory (29). This phase-specificity underscores the importance of integrating ferroptosis readouts with established fracture healing outcome measures including histomorphometry, micro-CT analysis, and biomechanical testing (84, 85).

Translating these experimental readouts to clinical settings remains challenging, as no validated clinical test for ferroptosis currently exists (86). Systemic iron status (serum ferritin, transferrin saturation, TIBC) and oxidative stress biomarkers (plasma MDA, F2-isoprostanes, 4-HNE adducts) are clinically available but reflect systemic rather than local fracture-site conditions. Site-specific approaches such as MRI T2* tissue iron mapping and immunohistochemical detection of GPX4, ACSL4, and 4-HNE in surgical specimens provide tissue-level evidence but require invasive sampling. Candidate circulating ferroptosis biomarkers including oxidized phosphatidylethanolamine species remain investigational without established reference ranges. This translational gap represents a critical priority for future research.

## 3 Trauma-associated microenvironment and ferroptotic vulnerability

### 3.1 The post-traumatic microenvironment as a ferroptosis-permissive niche

Orthopedic trauma generates a complex, evolving microenvironment with multiple ferroptosis-relevant features (87). The immediate consequence of fracture is hemorrhage and hematoma formation, which introduces substantial quantities of hemoglobin and heme-bound iron into the injury site. As erythrocytes lyse and macrophages engulf cellular debris, heme is catabolized by HO-1, releasing free iron that expands the local labile iron pool (88, 89). This iron flux, while necessary for subsequent reparative processes, creates conditions favoring Fenton chemistry and lipid peroxidation when antioxidant defenses are overwhelmed (90, 91).

Simultaneously, the inflammatory response mobilizes neutrophils and macrophages that generate ROS as part of their antimicrobial and debris-clearing programs. While physiological ROS levels contribute to redox signaling essential for healing, excessive or prolonged oxidative stress—particularly in severe injury, infection, or compromised host conditions—can exceed cellular buffering capacity and drive pathological lipid peroxidation (92, 93). The temporal overlap of iron release and inflammatory ROS production creates a biochemically synergistic environment for ferroptosis initiation.

Ischemia–reperfusion injury adds another layer of oxidative insult. Traumatic disruption of local vasculature causes tissue hypoxia, followed by ROS surge upon reperfusion as oxygen becomes available to partially reduced electron carriers (94). This fluctuating oxygen tension, combined with iron availability and inflammatory mediators, further amplifies lipid peroxidation potential (95, 96) In reconstruction contexts involving staged procedures—such as the induced membrane technique or serial debridement for infected defects—repeated surgical insults may perpetuate this cycle of ischemia–reperfusion and oxidative stress (97, 98).

Finally, the presence of orthopedic implants and biomaterials introduces additional considerations. Metal ion release and wear particle generation can perturb local redox balance, while certain biomaterial surfaces may influence macrophage polarization and inflammatory persistence (99–101). These factors are particularly relevant in compromised healing scenarios where prolonged implant–tissue interaction occurs.

### 3.2 A Phase-specific framework for ferroptosis in compromised bone healing

**FIGURE 1:**
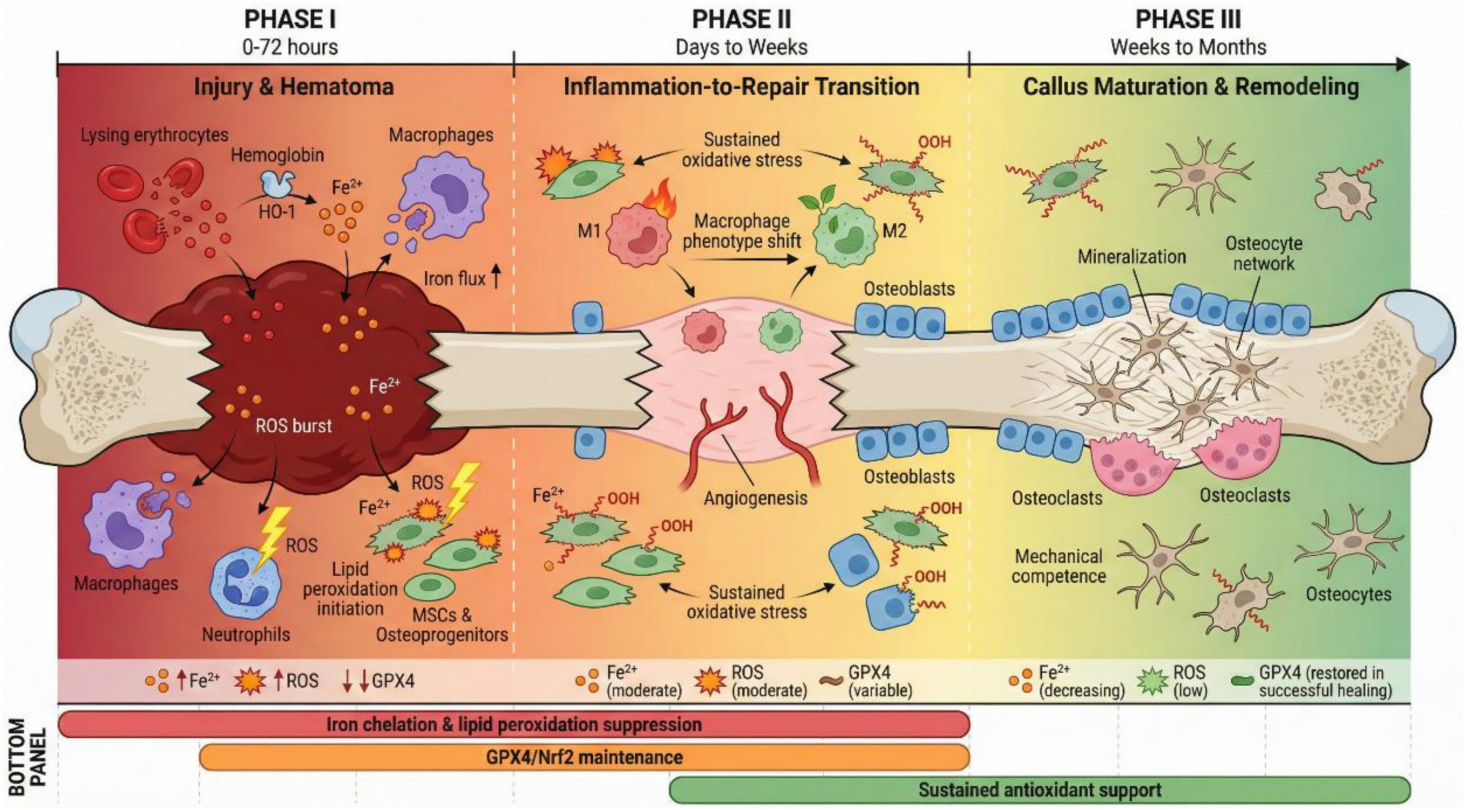
Phase-specific ferroptosis framework in trauma-compromised bone healing. Schematic illustration of ferroptosis-permissive features across fracture healing phases. Phase I (injury/hematoma): heme/iron flux, reactive oxygen species (ROS) burst, early lipid peroxidation threaten mesenchymal stromal/stem cells (MSCs) and osteoprogenitors; Phase II (inflammation-to-repair transition): sustained oxidative stress and macrophage dysregulation impair angiogenesis and osteogenic commitment; Phase III (callus maturation/remodeling): persistent ferroptotic pressure compromises mineralization and osteocyte network integrity, contributing to delayed union or non-union. Key intervention windows for lipid peroxidation suppression, iron chelation, and GPX4/Nrf2-targeted cytoprotection are indicated. Approximate timelines for each phase are indicated in the figure: Phase I (0–72 h post-injury), Phase II (days to weeks), and Phase III (weeks to months). These timelines are approximate and may vary depending on species, fracture type, fixation method, and host factors. Created with BioRender.com.

Fracture healing proceeds through overlapping phases—inflammation, repair (including soft and hard callus formation), and remodeling—each with distinct cellular composition, metabolic demands, and microenvironmental characteristics. We propose that ferroptotic vulnerability varies across these phases, with implications for both pathophysiology and therapeutic intervention (Figure 1).

During Phase I (hours to days), the acute injury and hematoma phase, iron/ROS flux is maximal. Osteogenic progenitors, MSCs, and osteoblasts at fracture ends are exposed to high concentrations of heme-derived iron and inflammatory ROS, potentially pushing vulnerable cells toward lipid peroxidation thresholds (102, 103). Early ferroptotic attrition of these critical effector populations may compromise the subsequent regenerative response.

In Phase II (days to weeks), the inflammation-to-repair transition and early angiogenesis phase, the balance between pro-inflammatory and pro-resolving programs determines healing trajectory (2, 104). If ferroptosis depletes osteogenic progenitors or skews macrophage phenotypes toward persistent M1 polarization, the inflammatory resolution and vascular ingrowth essential for callus formation may be delayed (105, 106). Macrophages themselves are subject to ferroptosis regulation, and their phenotypic plasticity may be influenced by local iron and lipid peroxidation status (107, 108). Importantly, macrophage polarization is a fundamental determinant of bone healing outcomes independent of ferroptosis: pro-inflammatory M1 macrophages dominate the early inflammatory phase and are essential for debris clearance and immune defense, while anti-inflammatory M2 macrophages promote angiogenesis, MSC recruitment, and osteogenic differentiation during the reparative phase (104–106). The timely transition from M1 to M2 polarization is critical for successful healing, and disruption of this switch is a recognized feature of impaired fracture repair (104).

Phase III (weeks to months) encompasses callus maturation, mineralization, and remodeling. Persistent oxidative stress and ongoing ferroptosis may impair osteoblast function, disrupt osteocyte network establishment, and compromise the mechanical competence of regenerating bone. In non-union, this phase fails to progress normally, and the tissue often exhibits signatures of sustained inflammation and impaired osteogenic differentiation (6).

The intersection of ferroptosis and macrophage polarization represents a critical but incompletely characterized axis in bone healing biology. Even independent of ferroptosis, macrophage polarization exerts profound effects on the bone healing microenvironment: M1 macrophages produce pro-inflammatory cytokines (TNF-α, IL-1β, IL-6) that, while necessary for initial immune responses, inhibit osteogenic differentiation when sustained; conversely, M2 macrophages secrete anti-inflammatory and pro-regenerative factors (IL-10, BMP-2, TGF-β) that support angiogenesis and bone formation (104–106). Ferroptosis intersects with this polarization landscape through multiple mechanisms. First, the iron and lipid peroxidation conditions that drive ferroptosis simultaneously influence macrophage polarization state: elevated intracellular iron and ROS favor M1 polarization, while antioxidant signaling through Nrf2 promotes M2 phenotype (107, 108). Second, ferroptotic macrophages have been shown to release damage-associated molecular patterns (DAMPs) and pro-inflammatory mediators—notably TNF-α—that suppress MSC osteogenic differentiation through paracrine mechanisms (109). Third, ferroptosis inhibition can shift macrophage polarization from M1 toward M2, thereby simultaneously reducing inflammatory damage and promoting a pro-regenerative microenvironment (109). This dual relationship implies that ferroptosis and macrophage polarization are not independent phenomena but rather form a self-reinforcing circuit: iron/ROS excess promotes both M1 polarization and ferroptotic vulnerability, while ferroptotic cell death further amplifies inflammation and impairs M1-to-M2 transition. Breaking this circuit through targeted ferroptosis inhibition may therefore yield benefits beyond simple cytoprotection, offering a means to restore the macrophage phenotypic switch essential for bone healing progression. The specific evidence supporting this crosstalk in infected and inflammatory bone healing contexts is discussed in detail in section “4.3 Infected bone defects: ferroptosis at the intersection of infection, immunity, and osteogene.”

This phase-specific framework provides a conceptual scaffold for interpreting ferroptosis evidence across different compromised healing phenotypes and for designing temporally targeted interventions.

## 4 Evidence linking ferroptosis to compromised bone healing phenotypes

**FIGURE 2:**
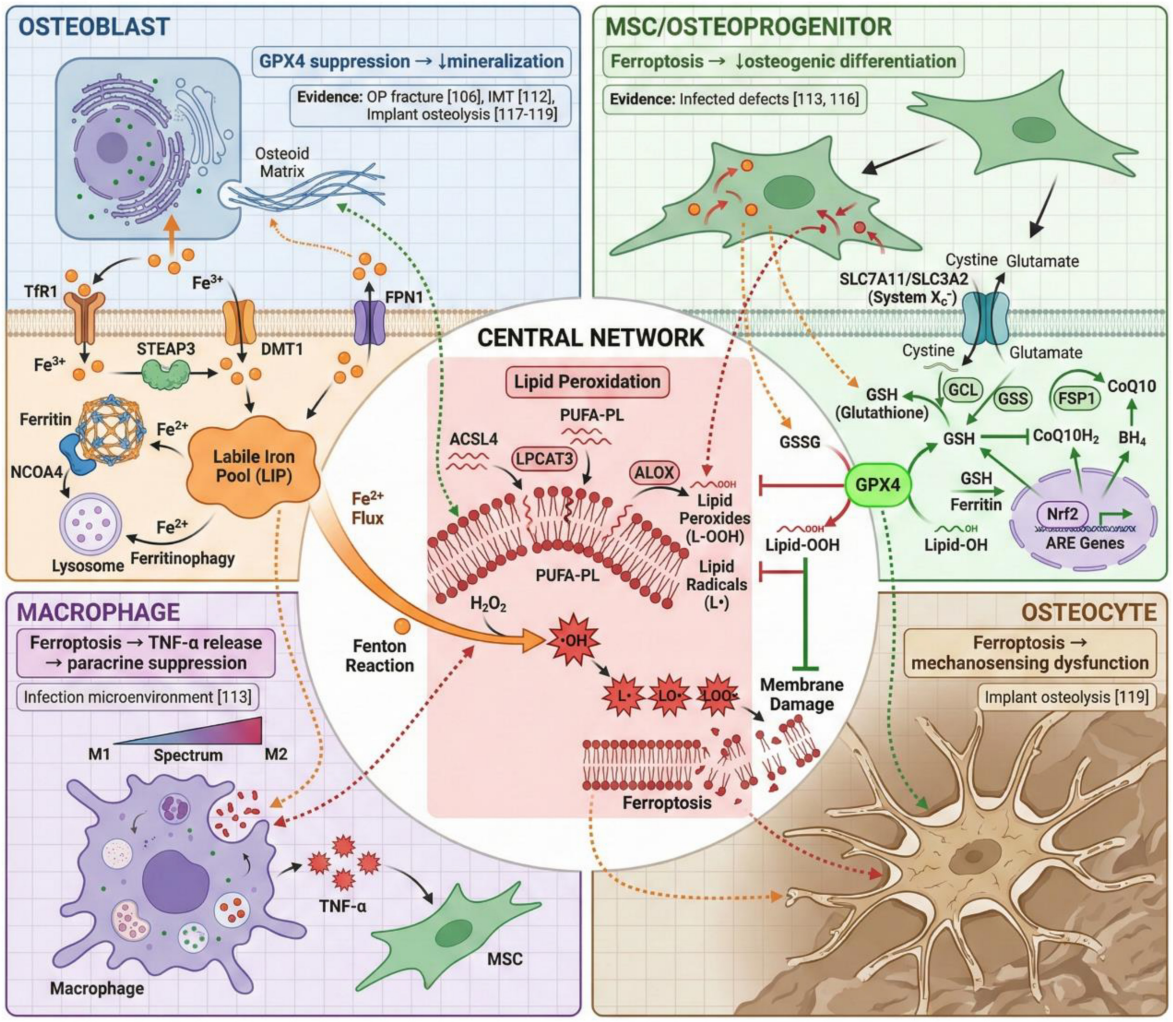
Ferroptosis regulatory network mapped to bone regeneration cell types. Central panel depicts the three core modules of ferroptosis: iron metabolism, lipid peroxidation, and antioxidant defense (GPX4/Nrf2 axis). Surrounding panels illustrate cell type-specific consequences—osteoblast mineralization defects, mesenchymal stromal/stem cells (MSC) differentiation impairment, macrophage-mediated TNF-α paracrine suppression of osteogenesis, and osteocyte mechanosensing dysfunction. Reference numbers indicate supporting original studies. ACSL4, acyl-CoA synthetase long-chain family member 4; ARE, antioxidant response element; CoQ10, coenzyme Q10; DMT1, divalent metal transporter 1; FPN1, ferroportin 1; FSP1, ferroptosis suppressor protein 1; GCL, glutamate-cysteine ligase; GPX4, glutathione peroxidase 4; GSH, glutathione; GSS, glutathione synthetase; LIP, labile iron pool; LPCAT3, lysophosphatidylcholine acyltransferase 3; NCOA4, nuclear receptor coactivator 4; Nrf2, nuclear factor erythroid 2-related factor 2; PUFA-PL, polyunsaturated fatty acid-containing phospholipid; STEAP3, six-transmembrane epithelial antigen of prostate 3; TfR1, transferrin receptor 1. Created with BioRender.com.

This section reviews original studies investigating the role of ferroptosis in specific clinical scenarios of compromised bone healing. We organize the discussion around clinically relevant phenotypes, summarizing for each the characteristic microenvironmental features, the ferroptosis-related mechanisms identified, and the therapeutic implications for reconstruction. Representative studies are presented in Table 1, and key regulatory networks mapped to bone cell types are illustrated in Figure 2.

**TABLE 1:** Representative original studies linking ferroptosis to compromised bone healing phenotypes.

Clinical scenario	References	Model system	Cell type(s)	Ferroptosis-related findings	Key ferroptosis marker(s) assessed	Intervention strategy	Main outcome
Osteoporotic fracture healing	Xiang et al. (110)	Rat OP fracture; osteoblast assays	Osteoblasts	Lipid peroxidation; GPX4/SLC7A11; Nrf2/HO-1	GPX4, SLC7A11, lipid peroxidation, Nrf2/HO-1	Icariin	Improved OP fracture healing
OP defect (injectable graft)	Huang et al. (111)	OP defect; PDT-TCP-SE with SeNPs	BMSCs	Sirt1/Nrf2/GPX4 axis	Sirt1, Nrf2, GPX4	Selenium nanoparticle graft	Reduced oxidative stress; improved regeneration
OP defect (hydrogel)	Ye et al. (112)	OP defect; Tremella hydrogel	Osteoblast-lineage	Iron overload/ferroptosis	Iron levels, ROS, ferroptosis markers	Dual anti-ferroptosis + OGP release	Rebalanced OP microenvironment
OP defect (iron overload)	Lin et al. (113)	Iron-overload OP defect; CS/HA hydrogel	Osteoblast-lineage	Iron overload → ROS → ferroptosis	Iron, ROS, lipid peroxidation	Iron chelation + ROS-responsive	Improved repair under iron overload
IMT grafting zone	Li et al. (114)	IMT grafting model	Osteoblasts	Iron overload; NRF2/ARE axis	NRF2, ARE, iron levels	NRF2/ARE activation	Enhanced IM osteogenesis
Infected bone defect	Yuan et al. (115)	Infected defect; 3D-printed scaffold	BMSCs	TLR → IRF7 → ferroptosis	TLR, IRF7, ROS, BMSC viability	ROS-responsive antibacterial scaffold	Improved infected defect repair
Bone infection microenvironment	Liu et al. (109)	Rat infection; scRNA-seq	Macrophages; MSCs	↑Acsl4/Lpcat3; ↓Gpx4; M1 polarization	Acsl4, Lpcat3, Gpx4, lipid ROS	Ferroptosis inhibition	Reduced inflammation; improved osteogenesis
Complex defect (Mg scaffold)	Zhao et al. (116)	3D-printed Mg-hydrogel scaffold	Osteogenic cells	Ferroptosis regulatory function	Lipid peroxidation, cell viability	Dual-layer Mg–hydrogel design	Enhanced regeneration
Defect repair (cell therapy)	Xu et al. (117)	Rat calvaria defect; miR-181a MSCs	MSCs	TP53/SLC7A11 axis	TP53, SLC7A11, lipid ROS	miR-181a overexpression	Accelerated defect repair
Implant osteolysis (CoCrMo)	Xu et al. (118)	Peri-implant osteolysis	Osteoblasts	Nrf2-ARE dysregulation	Nrf2, ARE, GPX4	Nrf2-ARE modulation	Reduced osteolysis
Implant osteolysis (Ti)	Liu et al. (119)	Wear particle osteolysis	Osteoblasts	GPX4 suppression	GPX4, lipid peroxidation	Urolithin A (GPX4 activator)	Rescued osteogenesis
Implant osteolysis (UHMWPE)	Ogawa et al. (120)	Murine osteolysis; clinical samples	Osteocytes; osteoblasts	ROS/lipid peroxidation	ROS, lipid peroxidation, 4-HNE	DFO; ferrostatin-1	Reduced inflammatory osteolysis
Non-union (bioinformatics)	Yu et al. (121)	Bioinformatics + MR	Gene-level inference	PTGS2/PRKCA/MAPK14	PTGS2, PRKCA, MAPK14	Computational (hypothesis-generating)	Proposed diagnostic targets

### 4.1 Osteoporotic fractures and bone defects

Osteoporotic fractures and bone defects are characterized by diminished osteogenic capacity, impaired angiogenesis, and compromised antioxidant reserve (122, 123). Aging and estrogen deficiency promote systemic iron accumulation, elevate oxidative stress in bone tissue, and suppress expression of antioxidant enzymes including GPX4 (16, 22). These alterations establish a baseline susceptibility to ferroptosis that may be further amplified by traumatic injury or surgical intervention.

In a rat osteoporotic fracture model, Xiang et al. demonstrated that icariin, a flavonoid glycoside with antioxidant properties, promoted callus formation and facilitated the transition from fibrous to osseous tissue (110). Mechanistic analyses revealed that icariin attenuated lipid peroxidation and preserved GPX4/SLC7A11 expression in osteoblasts, with the Nrf2/HO-1 axis implicated as a key regulatory pathway. This study establishes a direct link between osteoblast ferroptosis modulation and fracture healing outcomes in the osteoporotic setting.

Several recent studies have explored ferroptosis-targeted biomaterial strategies for osteoporotic bone defect reconstruction. Huang et al. developed an injectable selenium nanoparticle-integrated bone graft substitute (PDT-TCP-SE) that protected BMSCs from erastin-induced ferroptosis through activation of the Sirt1/Nrf2/GPX4 pathway (111). Application of this material in osteoporotic defect models resulted in reduced oxidative stress and enhanced bone regeneration. Ye et al. engineered a Tremella-derived injectable hydrogel (OGP@DTrep) incorporating dual anti-ferroptosis mechanisms coupled with ROS-responsive osteogenic peptide release (112). This system effectively rebalanced the osteoporotic microenvironment and promoted osteoregeneration by concurrently addressing iron overload and oxidative stress. Targeting the specific challenge of iron overload prevalent in elderly osteoporotic patients, Lin et al. designed a chitosan/hyaluronic acid hydrogel with chromium picolinate that provided synergistic iron chelation and ROS-responsive degradation (113). This approach demonstrated improved defect repair under iron overload conditions, underscoring the therapeutic potential of addressing upstream iron dysregulation.

These studies collectively indicate that bone injuries occurring under osteoporotic conditions exhibit heightened susceptibility to ferroptosis-mediated impairment of regeneration, likely due to the compounded effects of pre-existing iron dysregulation, diminished antioxidant reserves, and trauma-induced oxidative insult. Interventions targeting the iron/ROS/GPX4 axis can substantially improve regenerative outcomes in both fracture and defect contexts by addressing these converging vulnerabilities.

### 4.2 Induced membrane technique: ferroptosis in staged reconstruction

The Masquelet induced membrane technique (IMT) utilizes a temporary cement spacer to generate a vascularized biological membrane that subsequently supports autograft incorporation in segmental bone defects (97). The membrane’s osteogenic and angiogenic properties critically influence reconstruction success, yet the molecular determinants of membrane quality remain incompletely characterized.

Li et al. investigated ferroptosis in the IMT grafting zone and found that iron overload was associated with osteoblast ferroptosis, while NRF2/ARE axis activation conferred protection and enhanced membrane osteogenic capacity (114). These findings suggest that the induced membrane functions not merely as a mechanical chamber but as an oxidative stress-regulated niche where ferroptosis may compromise osteoblast viability and function. From a clinical perspective, IMT involves repeated surgical interventions—spacer insertion, membrane maturation, and grafting—each potentially introducing oxidative insult. The data from this study suggest that ferroptosis inhibition during membrane maturation and early graft incorporation may represent a rational adjunctive strategy to optimize reconstruction outcomes.

### 4.3 Infected bone defects: ferroptosis at the intersection of infection, immunity, and osteogenesis

Fracture-related infection and chronic osteomyelitis generate a microenvironment characterized by bacterial products, sustained inflammation, biofilm-associated oxidative stress, and elevated ROS. This hostile milieu compromises MSC survival and osteogenic differentiation while simultaneously requiring robust immune function for infection control—a therapeutic challenge that necessitates careful balancing of competing demands.

Yuan et al. developed an integrated approach combining a ROS-responsive antibacterial and anti-ferroptotic 3D-printed scaffold with modified BMSCs (115). Their investigations revealed that bacterial stimulation triggered BMSC ferroptosis through innate immune signaling cascades (TLR → IRF7), and that the scaffold system preserved BMSC viability and osteogenic capacity while maintaining antibacterial efficacy. This work identifies ferroptosis as a therapeutically addressable barrier to regeneration under infection-associated stress and demonstrates that effective intervention can simultaneously achieve infection control and cellular protection.

Liu et al. provided complementary mechanistic insights through integrated single-cell and bulk RNA-seq analysis of bone infection models (109). Their analysis identified macrophage ferroptosis as a pathological event in the infectious inflammatory microenvironment, characterized by elevated Acsl4/Lpcat3 expression, suppressed Gpx4, and increased lipid peroxidation. Notably, ferroptotic macrophages exhibited M1 polarization and inhibited MSC osteogenic differentiation through paracrine mechanisms. Ferroptosis inhibition shifted macrophage phenotype toward M2 and rescued MSC osteogenesis. These findings reveal a previously unrecognized ferroptosis-mediated crosstalk between immune and osteogenic compartments, representing a non-cell-autonomous mechanism whereby ferroptotic cell death in macrophages compromises regenerative function in MSCs through paracrine signaling. As discussed in section “3.2 A Phase-specific framework for ferroptosis in compromised bone healing” (Ferroptosis–Macrophage Polarization Crosstalk), the self-reinforcing circuit between ferroptosis and M1 polarization provides a mechanistic framework for understanding how infection-driven oxidative stress simultaneously amplifies inflammatory damage and suppresses osteogenesis. Targeting macrophage ferroptosis may therefore simultaneously attenuate this inflammatory circuit and establish a more permissive environment for bone regeneration.

### 4.4 Biomaterial-based ferroptosis modulation in bone defect repair

Beyond the specific clinical scenarios discussed above, recent studies have explored ferroptosis-targeted biomaterial strategies for general bone defect repair. Zhao et al. developed a biomimetic 3D-printed magnesium scaffold impregnated with hydrogel that incorporated ferroptosis regulatory function (116). The dual-layer Mg–hydrogel design attenuated lipid peroxidation and mitigated ferroptosis-associated cellular stress while promoting bone regeneration. This approach leverages the inherent biodegradability and osteogenic properties of magnesium while addressing the potential for ROS generation during degradation. The integration of anti-ferroptotic function into structural biomaterials represents an important design principle: rather than requiring separate pharmacological intervention, the scaffold itself provides both mechanical support and biochemical protection against ferroptosis-mediated cellular attrition.

### 4.5 Cell engineering approaches to ferroptosis resistance

Complementary to biomaterial-based strategies, cell engineering approaches offer the possibility of programming intrinsic ferroptosis resistance into therapeutic cell populations. Xu et al. engineered MSCs with miR-181a overexpression and delivered them via nano-hydroxyapatite/collagen scaffolds (117). miR-181a suppressed ferroptosis through the TP53/SLC7A11 axis, and the engineered MSCs demonstrated enhanced survival and osteogenic capacity in rat calvarial defects. This study illustrates that ferroptosis resistance can be genetically programmed into therapeutic cell populations to augment their regenerative efficacy in oxidatively challenging microenvironments. From a translational perspective, cell engineering approaches may be particularly valuable for severe or recalcitrant defects where the local microenvironment is expected to impose sustained oxidative stress that exceeds the protective capacity of biomaterial-mediated antioxidant delivery alone.

### 4.6 Implant-associated osteolysis: ferroptosis as a mechanism of wear particle-induced bone loss

Although not a fracture healing scenario *per se*, implant-associated osteolysis shares pathophysiological features with compromised bone healing: chronic inflammation, oxidative stress, and impaired bone cell function. Wear particles from orthopedic implants generate ROS, activate inflammatory cascades, and create conditions that may promote ferroptosis in bone-forming cells.

Three recent studies have directly investigated ferroptosis in periprosthetic osteolysis. Xu et al. demonstrated that CoCrMo nanoparticles induced osteoblast ferroptosis through dysregulation of Nrf2-ARE signaling, contributing to peri-implant bone loss (118). Liu et al. identified urolithin A as a small molecule capable of enhancing GPX4 activity and rescuing osteoblast ferroptosis induced by titanium nanoparticles, thereby attenuating periprosthetic osteolysis (119). Ogawa et al. extended these observations to ultra-high-molecular-weight polyethylene debris, detecting ferroptosis markers in osteocytes and osteoblasts in both murine models and clinical specimens; both deferoxamine and ferrostatin-1 reduced inflammatory osteolysis in their experimental system (120).

These studies establish that wear particle-induced osteolysis involves ferroptotic cell death in bone-forming cells, identifying ferroptosis inhibition as a potential therapeutic avenue for this challenging clinical problem. The mechanistic parallels with compromised fracture healing—iron/ROS excess, osteoblast vulnerability, and GPX4/Nrf2 as therapeutic targets—reinforce the broader applicability of ferroptosis biology across orthopedic pathologies.

### 4.7 Non-union: bioinformatic exploration of ferroptosis-related molecular signatures

Non-union represents the terminal outcome of failed fracture repair, encompassing clinically and mechanistically heterogeneous conditions (10). A significant obstacle to therapeutic development is the limited availability of human tissue-level molecular data that might link non-union pathophysiology to specific cell death pathways.

Yu et al. conducted a bioinformatics analysis intersecting non-union-associated gene expression signatures with ferroptosis-related gene sets, constructed diagnostic prediction models, and employed Mendelian randomization to explore potential causal relationships (121). Their analysis implicated PTGS2, PRKCA, and MAPK14 as ferroptosis-associated genes with diagnostic potential, and identified trace elements including vitamin E and potassium as potentially protective factors. While computational approaches of this nature require cautious interpretation and cannot establish causality, they generate testable hypotheses and highlight directions for future investigation. The convergence of transcriptomic data with ferroptosis-focused computational modeling supports the biological plausibility of ferroptosis involvement in non-union, while emphasizing the need for mechanistic validation in experimental models and prospective clinical studies. Similar multilevel bioinformatic approaches integrating transcriptomic, proteomic, and single-cell data have been applied to identify cell death-related molecular signatures in other musculoskeletal disorders, further supporting the broader relevance of regulated cell death pathways in skeletal pathology (124).

### 4.8 Summary and implications for reconstruction strategy

The studies reviewed in this section reveal several recurring themes across diverse compromised healing phenotypes. The post-traumatic and pathological microenvironments consistently exhibit ferroptosis-permissive characteristics—iron dysregulation, ROS excess, and antioxidant insufficiency—that vary in magnitude and duration but converge on similar cellular vulnerabilities. Osteoblasts, MSCs, and their progenitors emerge as principal ferroptosis targets whose attrition or functional impairment compromises regenerative capacity; macrophages constitute an additional important target population whose ferroptosis status shapes the broader regenerative microenvironment through paracrine and immunomodulatory mechanisms. Across intervention studies, the GPX4 and Nrf2 axes consistently emerge as therapeutically accessible regulatory nodes, whether targeted through small molecules, biomaterial design, or genetic engineering. In infected contexts, successful strategies demonstrate that ferroptosis inhibition must preserve antimicrobial function, emphasizing microenvironmental rebalancing rather than indiscriminate ROS suppression.

## 5 Therapeutic strategies and clinical integration

### 5.1 Intervention modules

Ferroptosis can be therapeutically targeted at multiple regulatory nodes, from upstream iron dysregulation to downstream lipid peroxidation and antioxidant failure. These interventions fall into several conceptually distinct modules (Table 2).

**TABLE 2:** Intervention modules for ferroptosis modulation in compromised bone healing.

Module	Target	Rationale	Implementation	Best context	References
Iron chelation + ferritin promotion	Labile iron pool	Reduces Fenton chemistry while maintaining iron homeostasis	IBDS nanoparticles; ROS-responsive hydrogels	Iron overload; OP; smoking	(112, 113, 120, 125)
Lipid peroxidation suppression	Lipid radicals	Terminates chain reactions	Fer-1/Lip-1 in biomaterials	Early phase; implant osteolysis	(79, 120, 126–128)
GPX4/Nrf2 reinforcement	Antioxidant defense	Restores terminal protection	Small molecules; SeNPs; icariin	Broad applicability; IMT	(110, 111, 114, 119)
Macrophage ferroptosis modulation	TNF-α paracrine axis	Interrupts immune-osteogenic crosstalk	NRF2/FSP1 activation	Infected/inflammatory	(109)
Cell engineering	Intrinsic MSC protection	Programs ferroptosis resistance	miR-181a overexpression	Cell therapy	(116, 117)
Multifunctional platforms	Multiple nodes	Integrates structure with protection	Mg-hydrogel; SeNP scaffolds	Complex defects	(111, 112)

Iron homeostasis restoration addresses the root driver of ferroptosis—labile iron pool expansion and subsequent Fenton chemistry. Traditional chelators such as deferoxamine have demonstrated efficacy in reducing wear particle-induced osteolysis (120), and this principle has been extended to biomaterial design through ROS-responsive hydrogels with intrinsic chelating capacity (112, 113). However, simply removing iron is not always sufficient and may even be counterproductive. A more sophisticated approach combines chelation with enhanced iron sequestration through ferritin upregulation (125). The iron balance dual-drive strategy (IBDS) exemplifies this concept: Deferiprone-conjugated polyamidoamine dendrimer with triphenylphosphonium mitochondria-targeting moiety (DFP-PAMAM-TPP) nanoparticles not only chelate mitochondrial iron but also promote ferritin synthesis via P5CS-mediated pathways, thereby avoiding the toxicity associated with excessive chelation while maintaining physiological iron homeostasis (125).

Lipid peroxidation suppression targets the propagation phase of ferroptotic damage. Radical-trapping antioxidants such as ferrostatin-1 and liproxstatin-1 intercept lipid radicals before they can amplify into destructive chain reactions (79, 126). These agents have served as positive controls across bone healing studies, confirming ferroptosis involvement when their application rescues cell viability or regenerative outcomes. For clinical translation, the key challenge is achieving adequate local concentrations without systemic interference with physiological redox signaling. Incorporation into degradable biomaterials with controlled release kinetics offers a practical solution compatible with surgical workflows (127, 128).

GPX4/Nrf2 axis reinforcement represents the most extensively validated therapeutic approach. Multiple strategies converge on this pathway: small molecules such as urolithin A directly promote GPX4 activity (119); selenium nanoparticle-integrated biomaterials activate the Sirt1/Nrf2/GPX4 cascade (111); natural compounds including icariin and dimethyl fumarate engage Nrf2/HO-1 and NRF2/ARE transcriptional programs (110, 114); and genetic approaches such as miR-181a overexpression target the TP53/SLC7A11 axis (117). The appeal of Nrf2 as a therapeutic target lies in its coordinated regulation of GPX4, glutathione synthesis, and iron-handling genes—activating Nrf2 simultaneously reinforces multiple defense mechanisms. One caveat deserves attention: HO-1, a canonical Nrf2 target, releases free iron from heme degradation, potentially counteracting anti-ferroptotic benefits if iron-buffering capacity is insufficient (66, 129). Optimal outcomes likely require balanced activation of both antioxidant and iron-sequestration arms of the Nrf2 program.

Multifunctional biomaterial platforms integrate several of these modules into single, clinically deployable systems. Magnesium-hydrogel scaffolds combine structural support with ferroptosis regulation (116); selenium-integrated bone substitutes provide both osteoconductivity and antioxidant protection (111); ROS-responsive hydrogels offer staged release capabilities that can be matched to healing phase requirements (112). This integration is particularly attractive for translation because it leverages existing surgical workflows without requiring separate pharmacological interventions. Notably, similar biomaterial-based strategies combining ferroptosis modulation with macrophage reprogramming have shown promise in other musculoskeletal contexts, including temporomandibular joint osteoarthritis (130).

### 5.2 Matching intervention to microenvironment

Different compromised healing phenotypes exhibit distinct iron/ROS profiles, and intervention strategies should be tailored accordingly rather than applied uniformly.

Osteoporotic and iron-overload contexts are characterized by chronic iron accumulation and diminished antioxidant reserve, creating baseline ferroptotic vulnerability that trauma or surgery can push past critical thresholds. Importantly, the iron dysregulation in these settings is not simply a matter of excess intake—it reflects accelerated ferritinophagy, the NCOA4-mediated autophagic degradation of ferritin that liberates stored iron into the labile pool (131). This process is driven by AMPK/ULK1 pathway activation in response to oxidative stress, and it creates a vicious cycle: released iron catalyzes Fenton reactions, the resulting ROS further activate AMPK, and ferritinophagy accelerates (131). Breaking this cycle requires more than iron chelation alone; strategies that promote ferritin synthesis—such as the IBDS approach—directly counteract the underlying ferritinophagy mechanism (125).

Infected bone defects present a fundamentally different challenge. Here, ROS are not merely pathological byproducts but essential weapons of host defense—neutrophils and macrophages generate oxidative bursts to kill bacteria (132, 133). Indiscriminate antioxidant therapy in this context risks compromising infection control, a potentially catastrophic trade-off. Recent mechanistic work has revealed an additional layer of complexity: infection triggers ferroptosis not only in osteogenic cells but also in macrophages, and ferroptotic macrophages release TNF-α that suppresses MSC osteogenesis through paracrine signaling (115). This creates a link between innate immune dysregulation and regenerative failure that was not previously appreciated. Successful intervention in infected contexts therefore requires selective targeting—interrupting macrophage ferroptosis to break the TNF-α paracrine loop while preserving the antimicrobial oxidative capacity needed for infection control. Dual-function scaffolds that provide initial antibacterial activity followed by transition to anti-ferroptotic support as infection resolves offer one rational approach (115).

Induced membrane technique grafting zones experience repeated oxidative insults from staged surgical procedures—spacer insertion, membrane maturation, and eventual grafting each introduce tissue trauma and associated iron/ROS flux. Evidence that NRF2/ARE activation protects osteoblasts and enhances membrane osteogenic capacity suggests that ferroptosis inhibition during membrane maturation and early graft incorporation may improve outcomes (114). From a translational perspective, IMT is attractive because its staged workflow provides natural intervention windows: anti-ferroptotic biomaterials could be incorporated at spacer removal without requiring fundamental changes to established protocols.

Non-union remains the least characterized scenario. Current evidence is limited to bioinformatic analyses implicating PTGS2, PRKCA, and MAPK14 as ferroptosis-associated genes with diagnostic potential (121). While the pathophysiological features of established non-union—persistent inflammation, impaired vascularization, compromised osteogenic function—are consistent with ferroptosis involvement, systematic molecular profiling of human non-union tissues is needed before specific intervention strategies can be rationally designed.

### 5.3 Timing considerations

Beyond spatial matching of intervention to microenvironment, temporal considerations also matter. During the early inflammatory phase (hours to days post-injury), iron and ROS flux peak as hematoma forms and inflammatory cells infiltrate; this window may be optimal for lipid peroxidation suppression and iron chelation to preserve the osteogenic progenitors needed for subsequent regeneration. During the transition to repair (days to weeks), maintaining GPX4/Nrf2 programs supports osteogenic differentiation and helps shift macrophage polarization toward pro-regenerative phenotypes. During callus maturation (weeks to months), sustained antioxidant support may optimize mineralization and mechanical competence. These temporal dynamics suggest that biomaterials with staged or sequential release capabilities—rather than bolus or continuous delivery—may achieve superior outcomes by matching intervention intensity to phase-specific needs (134, 135).

### 5.4 Design principles

Several principles emerge for integrating ferroptosis modulation into reconstruction practice: First, microenvironmental assessment should guide intervention selection. Iron-overload and ferritinophagy-dominant contexts warrant chelation combined with ferritin promotion; infected contexts require selective cellular protection with particular attention to macrophage-mediated paracrine effects on osteogenesis. Second, mechanistic pathway matching improves precision. Ferritinophagy-driven scenarios benefit from strategies targeting the NCOA4/AMPK/ULK1 axis; macrophage-mediated regenerative suppression indicates NRF2/FSP1 reinforcement and TNF-α pathway modulation. Third, local biomaterial-based delivery integrates anti-ferroptosis function with surgical workflows, avoiding the complexities of systemic pharmacotherapy while achieving adequate concentrations at the site of need. Fourth, ferroptosis modulation complements rather than replaces reconstruction fundamentals. Mechanical stability, vascular supply, and infection control remain essential; anti-ferroptosis strategies represent an additional therapeutic dimension that enhances outcomes when appropriately integrated with these established principles.

## 6 Conclusion and future perspectives

This review has examined ferroptosis as a mechanistic node connecting iron/ROS-perturbed post-traumatic microenvironments with impaired bone regeneration. Original studies across osteoporotic fractures and defects, smoking-related bone loss, induced membrane reconstruction, and infected bone defects identify ferroptosis as a biologically plausible and therapeutically addressable contributor to compromised healing. The cellular targets vary by context: osteoblasts and MSCs undergo ferroptosis in most scenarios; in infected and inflammatory settings, macrophage ferroptosis additionally influences the regenerative microenvironment through TNF-α-mediated paracrine suppression of MSC osteogenesis. The mechanistic pathways also differ—NCOA4-mediated ferritinophagy predominates in smoking-related contexts while NRF2/FSP1/ROS axis dysregulation characterizes infection-associated macrophage ferroptosis. These distinctions have therapeutic implications: the IBDS strategy targeting mitochondrial iron chelation with ferritin synthesis promotion addresses ferritinophagy-driven ferroptosis, while NRF2 activation or TNF-α neutralization may be more appropriate for macrophage-mediated scenarios.

Critical gaps remain. Human tissue evidence directly linking ferroptosis to healing failure is limited; molecular profiling of non-union and delayed union specimens is needed. Optimal intervention timing across healing phases has not been systematically defined. Additionally, the mechanistic pleiotropy of commonly used ferroptosis inhibitors warrants careful interpretation: deferoxamine, for example, functions not only as an iron chelator but also as a prolyl hydroxylase domain inhibitor that stabilizes HIF-1α and promotes angiogenesis (136, 137), making it difficult to attribute therapeutic benefits specifically to ferroptosis inhibition versus pro-angiogenic effects. Future experimental designs should incorporate pathway-specific controls and multiple orthogonal ferroptosis readouts to disentangle these contributions. The balance between ferroptosis inhibition and preservation of ROS-dependent antimicrobial defense requires continued investigation. Clinical translation will require prospective trials with endpoints meaningful to reconstructive practice. The IMT setting, with its staged surgical workflow and strong original evidence, may represent an early translational opportunity.
